# High incidence of decidualization failure in infertile women

**DOI:** 10.1002/rmb2.12580

**Published:** 2024-05-16

**Authors:** Isao Tamura, Yumiko Doi‐Tanaka, Akihisa Takasaki, Katsunori Shimamura, Toshihide Yoneda, Hitomi Takasaki, Amon Shiroshita, Taishi Fujimura, Yuichiro Shirafuta, Norihiro Sugino

**Affiliations:** ^1^ Department of Obstetrics and Gynecology Yamaguchi University Graduate School of Medicine Ube Japan; ^2^ Department of Obstetrics and Gynecology Saiseikai Shimonoseki General Hospital Shimonoseki Japan

**Keywords:** decidualization failure, early pregnancy loss, endometrial dating, endometrium, implantation

## Abstract

**Purpose:**

Decidualization is an important event for embryo implantation and successful pregnancy. Impaired decidualization leads to implantation failure and miscarriage. However, it is unclear how often decidualization failure occurs in infertile women. By analyzing the endometrium at late‐secretory phase, we investigated the incidence and pathogenesis of decidualization failure among infertile women.

**Methods:**

Endometrial dating was performed on the endometria obtained in the late‐secretory phase from 33 infertile women. Endometrial dating of more than 2 days delay was taken as an indication of decidualization failure. The expression of essential transcription factors for decidualization (FOXO1, WT1, and C/EBPβ) was examined by immunohistochemistry.

**Results:**

Among 32 cases, 20 cases (62.5%) showed decidualization failure. These patients tended to have a history of more frequent miscarriages than those without decidualization failure. The percentage of cells that immunostained positive for the expression of three transcription factors was significantly lower in the patients with decidualization failure than in those without decidualization failure. Serum progesterone levels measured in the mid‐ and late‐secretory phase were not significantly different between the cases with and without decidualization failure.

**Conclusions:**

The incidence of decidualization failure is high in infertile women.

## 1 INTRODUCTION

Implantation of the human embryo in the maternal endometrium is key to the establishment of pregnancy and requires a dialog between the embryo and the receptive endometrium. In humans, this process is initiated by progesterone from the corpus luteum and involves morphological and functional changes in the endometrial cells. Human endometrial stromal cells (ESCs) undergo cyclic changes during the menstrual cycle, including proliferation and differentiation that are controlled by estrogen and progesterone.^1^, ^2^, ^3^, ^4^, ^5^ Decidualization is one of these changes in which ESCs respond to progesterone, and become larger and rounder. This process begins in the late secretory phase of the menstrual cycle. During decidualization, a number of cellular functions are altered in ESCs with dramatic changes in gene expression, which are regulated by key transcription factors, such as CCAAT enhancer binding protein beta (C/EBPβ), forkhead box O1 (FOXO1), and WT1 transcription factor (WT1).^6^, ^7^, ^8^, ^9^, ^10^, ^11^, ^12^, ^13^, ^14^ Decidualization is believed to be critical for embryo implantation and the establishment of pregnancy because a defective decidualization response is associated with reproductive disorders such as implantation failure, miscarriage, and early pregnancy loss.^15^, ^16^, ^17^ Previous reports have suggested that proper decidualization helps to prevent the implantation of chromosomally abnormal embryos and subsequent miscarriages.^1^, ^15^, ^18^, ^19^ In addition, decidualized ESCs (dESCs) are reported to regulate the proliferation, migration, and differentiation of trophoblast cells,^20^, ^21^, ^22^, ^23^ indicating that decidualization contributes to the early stage of placentation. Despite the essential role of decidualization in the establishment and maintenance of pregnancy, it remains unclear how often infertile women suffer from impaired decidualization. In this study, by analyzing the histology of the endometrium obtained at the late‐secretory phase, we investigated the incidence of decidualization failure among a cohort of infertile women. Furthermore, we also examined the expression of key transcription factors involved in decidualization to see whether there are cellular dysfunctions in dESCs in patients with decidualization failure.

## 2 MATERIALS AND METHODS

### 2.1 Patients

This study was conducted in accordance with the Declaration of Helsinki, and approved by the Institutional Review Board of Saiseikai Shimonoseki General Hospital, and Yamaguchi University Hospital. Informed consent was obtained from all the patients in this study. Thirty‐two patients with regular menstrual cycles who visited Saiseikai Shimonoseki General Hospital for infertility were enrolled in this study. They agreed to undergo an endometrial biopsy at the late‐secretory phase. All the patients who were involved in this study allowed the researchers to use their medical record data for research in an unidentifiable manner. Written informed consent was obtained from all the patients prior to the sample collections.

### 2.2 Endometrial biopsy and progesterone measurement

All patients were monitored throughout a natural cycle, with a daily ultrasonographic scan from days 10 to 12 of the menstrual cycle until the diameter of the dominant follicle reached 20 mm. The day that the dominant follicle disappeared was considered the day of ovulation (post‐ovulation + 0, PO + 0). Endometrial biopsy was performed at PO + 11 ~ 13 using a sterile pipet. The tissues were fixed in formalin, embedded in paraffin, and cut into 3‐μm sections. The sections were used for hematoxylin and eosin (H&E) staining and immunohistochemistry. The serum progesterone and levels were examined at the mid‐secretory (PO + 6 ~ 8) and late‐secretory (PO + 11 ~ 13) phases.

### 2.3 Histologic endometrial dating

All H&E‐stained endometrial biopsies were analyzed in a blinded manner to evaluate endometrial dating and stromal development. The endometrial dating was verified according to the Noyes dating criteria.^24^ All endometrial dating was determined by one experienced histologist and two experienced gynecologists. Endometrial dating of more than 2 days delay was taken as an indication of decidualization failure.

### 2.4 Immunohistochemistry

Endometrial sections were immunohistochemistry stained for three transcription factors (FOXO1, WT1, and C/EBPβ). Among the 32 cases, nine cases without decidualization failure and 11 cases with decidualization failure were analyzed. Histofine Simple Stain MAX‐PO (R) (Nichirei Co Ltd) was used following the manufacturer's protocol as previously reported.^25^, ^26^, ^27^ In brief, the sections were incubated with anti‐FOXO1 (Abcam plc), anti‐WT1 antibodies (Abcam), and anti‐C/EBPβ (Santa Cruz Biotechnology, Inc) at 4°C overnight followed by incubation with the secondary antibodies. Negative control sections were incubated with normal rabbit serum. Peroxidase activity was visualized by incubating the sections with 3,3′‐diaminobenzidine‐4 HCl (Nacalai Co. Ltd.) in 0.05 M Tris–HCl buffer (pH 7.6) containing 0.01% H_2_O_2_ for 3 min. The sections were counterstained with Meyer's hematoxylin. The percentages of stromal cells positive for FOXO1, WT1, and C/EBPβ were quantitated by analyzing three random microscopic fields at a magnification of 400x, respectively, by two independent observers who were blinded to the diagnosis. The mean value from the two observers was used as the percentage of positive cells in each sample.

### 2.5 Statistical analysis

Chi‐squared tests or Mann–Whitney *U* tests were used to analyze differences between the two groups. All statistical analyses were performed using R (version 4.0.2, R Foundation for Statistical Computing, Vienna, Austria). Differences were considered significant at *p* < 0.05.

## 3 RESULTS

### 3.1 Incidence of decidualization failure

**FIGURE 1:**
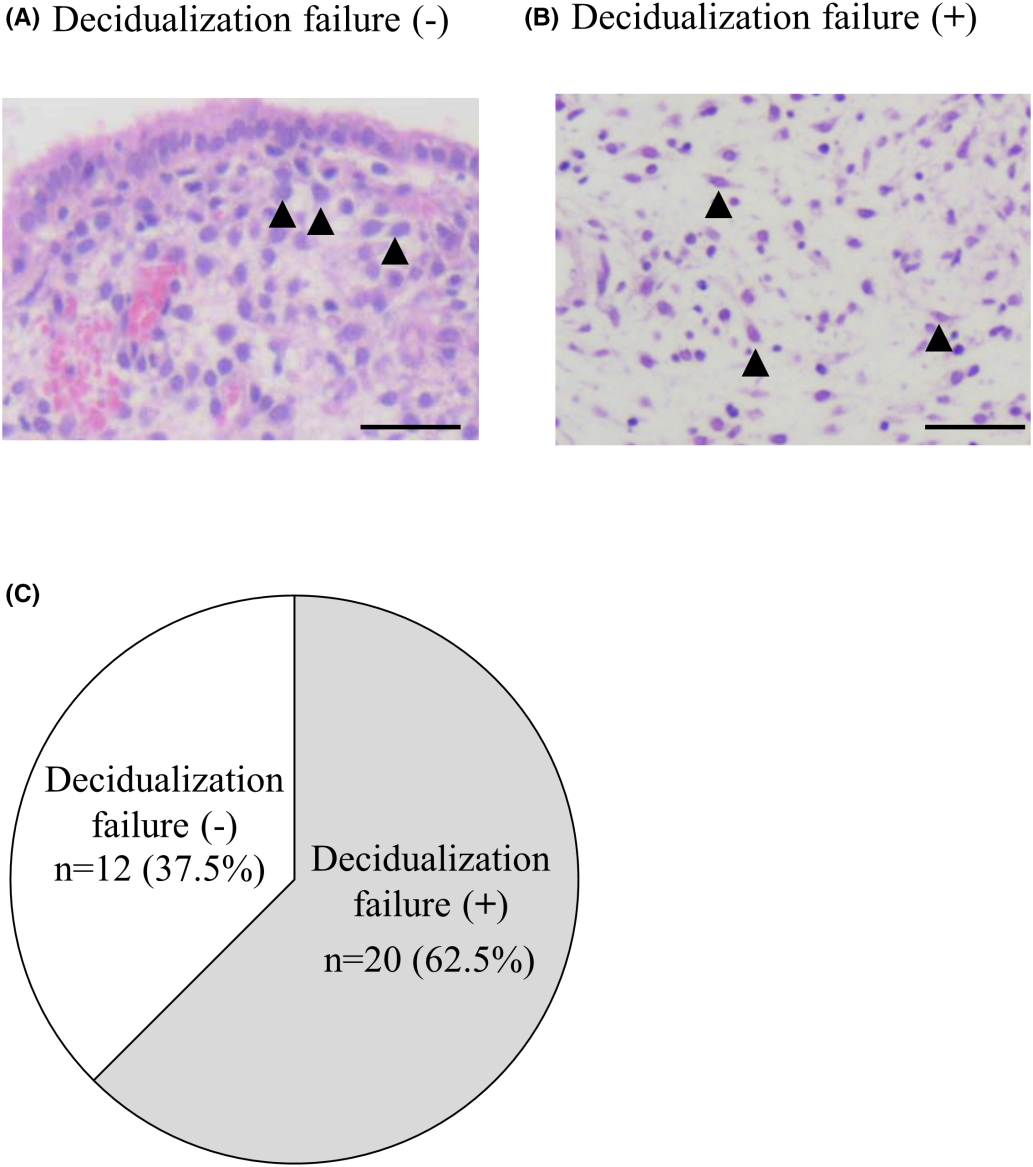
Histology and incidence of decidualization failure. Endometrial dating was performed on the endometrium obtained in the late‐secretory phase (PO + 11 ~ 13). Endometrial dating of more than 2 days delay was taken as an indication of decidualization failure. Representative images of the endometrium obtained at PO + 11 are shown. (A) Case without decidualization failure. Pre‐decidualized cells with large round morphology (arrowheads) were widely observed until the superficial layer of the endometrium with subtle stromal edema, which was determined as the histology of PO + 11. Scale bars, 50 μm. (B) Case with decidualization failure. The stromal cells showed spindle‐like morphology (arrowheads) and pre‐decidual cells were not observed. The stromal layer showed substantial edema. Scale bars, 50 μm. (C) Incidence of decidualization failure. Among 33 cases, 20 cases (62.5%) showed decidualization failure.

Endometrial dating was performed on endometria obtained in the late‐secretory phase (PO + 11 ~ 13). No notable changes occur in the epithelium during the mid‐ to late‐secretory phase endometrium, the evaluation of the epithelium is not useful for endometrial dating.^24^ Therefore, we did the endometrial dating according to the characteristics of the stroma as described below. Stromal edema is highest at the mid‐secretory phase (PO + 8) and gradually decreases toward the late secretory phase. ESCs at PO + 8 have small, dense naked nuclei with only filamentous cytoplasm. The spiral artery becomes prominent from PO + 9 and predecidual reaction (enlargement of cytoplasm and nuclei) is observed in ESCs surrounding the spiral arteries. The predecidual reaction progresses to the superficial layer of the endometrium at PO + 11. Leukocyte infiltration begins at PO + 12 and becomes more prominent at PO + 13. Endometrial dating was based on an overall assessment of the degree of these points. Endometrial dating of more than 2 days delay was taken as an indication of decidualization failure. Representative images of the endometrium obtained at PO + 11 are shown in Figure 1. In the endometrium shown in Figure 1A, pre‐decidualized cells with large round morphology (arrowheads) were widely observed in the endometrium. The stromal layer showed subtle edema. Endometrial dating was determined as PO + 11. Therefore, this case was diagnosed as a normal case without decidualization failure. On the other hand, in the endometrium shown in Figure 1B, stromal cells showed a spindle‐like morphology (arrowheads), and pre‐decidual cells were not observed. The stromal layer showed substantial edema. The histology was determined as PO + 8, which corresponds to the mid‐secretory phase. Therefore, this case was diagnosed as a case with decidualization failure. Among the 32 cases, 20 cases (62.5%) showed decidualization failure (Figure 1C). These results indicated that many infertile women show an insufficient decidual reaction, even at the late‐secretory phase. There were no significant differences in patient's age and complications between the cases with and without decidualization failure (Table 1). The patients with decidualization failure had experienced more pregnancies and miscarriages than those without decidualization failure, although the difference was not significant (Table 1). Especially, histories of multiple miscarriages were only found in the cases with decidualization failure (4 cases, 20%). Serum progesterone levels measured in the mid‐secretory phase (PO + 6 ~ 8) and late‐secretory phase (PO 11 ~ 13) were not significantly different between women with and without decidualization failure.

**TABLE 1:** Characteristics of patients.

	Decidualization failure (−) (*n* = 12)	Decidualization failure (+) (*n* = 20)
Patient age (mean ± SD)	34.9 ± 3.0	33.6 ± 5.5
BMI (mean ± SD)	21.9 ± 3.6	20.3 ± 2.2
Gravidity		
0 (%)	10 (83.3)	11 (55.0)
1 (%)	2 (16.7)	4 (20.0)
≧2 (%)	0	5 (25.0)
Parity		
0	12 (100)	18 (90.0)
1	0 (0)	2 (10.0)
History of miscarriages		
0 (%)	10 (83.3)	13 (65.0)
1 (%)	2 (16.7)	3 (15.9)
≧2 (%)	0	4 (20.0)
Patient's complications		
Myoma/adenomyosis (%)	2 (16.7)	2 (10.0)
Endometriosis (%)	1 (8.3)	1 (5.0)
Tubal factor (%)	0 (0)	1 (5.0)
Male factor (%)	1 (8.3)	3 (15.0)
Serum progesterone (ng/mL)		
Mid‐secretory phase	14.3 ± 6.6	15.0 ± 6.1
Late‐secretory phase	7.5 ± 4.8	8.9 ± 9.9

### 3.2 Impaired expression of transcription factors in ESCs with decidualization failure

**FIGURE 2:**
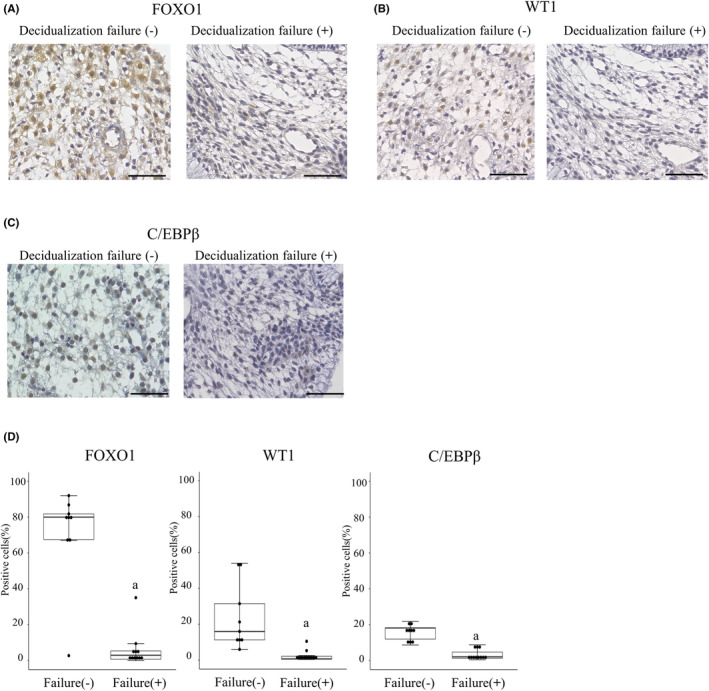
Expression of transcription factors in the late‐secretory phase endometrium with or without decidualization failure. Immunohistochemistry of three transcription factors (FOXO1, WT1, and C/EBPβ) was performed with endometrial sections obtained from nine cases without decidualization failure and 11 cases with decidualization failure. Representative images of immunohistochemistry of FOXO1 (A), WT1 (B), and C/EBPβ (C) in the endometrium with or without decidualization failure. Scale bars, 50 μm. (D) The percentage of stromal cells positive for FOXO1, WT1, and C/EBPβ were quantitated by analyzing three random microscopic fields at a magnification of 400×, respectively. Data for each case are shown with dot plots. Solid bars of boxes display the interquartile ranges (25%–75%) with an intersection as the median. The whiskers extending outside the boxes correspond to the lowest and highest data within a 1.5‐fold interquartile range from the upper and lower quartile. Mann–Whitney *U* test was applied to analyze the difference between the two groups. ^a^ *p* < 0.01 versus without decidualization failure.

During decidualization, dynamic changes in cellular function occur in ESCs, which are critical for successful implantation. The changes are regulated by several essential transcription factors for decidualization, FOXO1, WT1, and C/EBPβ.^6^, ^7^, ^8^, ^9^, ^10^, ^11^, ^12^, ^13^, ^14^ To examine cellular function of decidualized ESC, expressions of these transcription factors were immunohistochemically investigated. Their expressions were located in the nucleus, which is a characteristic of transcription factors. The percentage of cells in the stroma that were positive for these transcription factors was significantly lower in the patients with decidualization failure (Figure 2). These results suggest that ESCs in patients with decidualization failure have cellular dysfunctions. The reason for the low percentages of C/EBPβ‐positive cells, even in patients without decidualization failure, remains unclear. One possibility is that the antibodies used for immunohistochemistry had low reactivities.

## 4 DISCUSSION

Although previous reports suggested that some infertile women had impaired decidualization,^15^, ^16^, ^17^ it is unclear to what degree decidualization failure occurs in infertile women. The present study, for the first time, showed that infertile women have a high incidence of morphological and functional decidualization failure based on histological examinations of the endometrium. During decidualization, ESCs change their fibroblast‐like morphology to large round cells with changes in cellular function.^1^, ^3^, ^13^, ^28^, ^29^ The present study showed that decidual reactions were not morphologically sufficient in the ESCs of 62.5% infertile women. In addition, our results suggested that morphologically identified decidualization failure is accompanied by cellular dysfunction. Transcription factors, C/EBPβ, WT1, and FOXO1 contribute to decidualization through the alteration of many cellular functions, including cellular metabolism, angiogenesis, inflammation, immune system, response to oxidative stress, metabolisms, morphological change, cell migration, and cell proliferation.^6^, ^7^, ^8^, ^9^, ^10^, ^11^, ^12^, ^13^, ^14^, ^29^, ^30^, ^31^, ^32^, ^33^ In this study, endometria lacking morphological decidual change showed lower expression of these transcription factors. Taken together, decidualization failure is a pathological condition lacking normal morphological and functional decidualization status.

The high incidence of decidualization failure that we observed appears to be consistent with a study by Wilcox et al.^34^ In their study, urinary human chorionic gonadotropin (hCG) was measured using a highly sensitive assay system after the period of implantation in 221 healthy women, representing 221 menstrual cycles. An increase in the hCG levels was detected in 198 of these cycles. Of these, 43 cycles (22%) that were terminated before pregnancy were clinically confirmed by the commonly used urine hCG test. These results suggested that as many as 22% of cycles were not recognized as pregnancy and resulted in implantation failure or early pregnancy loss. In other words, pregnancy was not maintained after implantation in many cases. Considering that decidualization is observed in the late‐secretory phase endometrium, it is possible that impaired decidualization is associated with such implantation failure or early pregnancy loss.

Decidualization is induced by progesterone from the corpus luteum. Serum progesterone levels measured in the mid‐ and late‐secretory phase were not significantly different between women with and without decidualization failure. This suggests that decidualization failure was not due to the impaired progesterone secretion from the corpus luteum. Decidualization is induced by the activation of signal transduction pathways downstream of progesterone/progesterone receptor (PR).^28^ Transcription factors, C/EBPβ, WT1, and FOXO1 are involved in the activation of the signal transduction pathways downstream of PR.^1^, ^28^ Since expressions of these transcription factors were low in the ESCs of decidualization failure, the suppression of signaling pathways downstream of PR may be responsible for decidualization failure. On the other hand, it may be important to know the PR expression levels in the ESCs of decidualization failure. It is well known that PR expression in the endometrium is highest in the late proliferative phase, gradually decreases during the secretory phase after ovulation, and becomes very low in ESCs in the late‐secretory phase.^35^ Since we analyzed the endometrium from the late‐secretory phase in this study, very low expression of PR was expected. Therefore, we did not examine the PR expression in the late‐secretory phase endometrium. In addition, although PR expression in the endometrium from the late proliferative phase to the mid‐secretory phase may be important for the induction of decidualization, it is not practical to collect endometrial tissue twice: one to measure PR expression and one to evaluate decidualization. Future studies are needed to investigate how decidualization failure is caused or how the signaling pathways downstream of PR are suppressed in women with decidualization failure. It should also be noted that this study was based on a small sample size and used only immunohistochemistry. To obtain more solid evidence for decidualization failure, further studies are needed that analyze the endometrium with a large sample size and also use other techniques, such as single‐cell RNA‐sequence analysis.

In conclusion, the present study showed that infertile women with decidualization failure exist with a high incidence. In view of the importance of decidualization in establishment and maintenance of pregnancy, decidualization failure should not be overlooked in women with unexplained infertility, recurrent implantation failure, or recurrent miscarriage. However, there seem to be several issues to be solved for future clinical practice. How should we diagnose decidualization failure? When diagnosed, how should we treat decidualization failure? What is the cause of decidualization failure? Further studies will be needed to answer these questions.

## CONFLICT OF INTEREST STATEMENT

The authors declare no conflict of interest. Isao Tamura and Norihiro Sugino are Editorial Board members of Reproductive Medicine and Biology and co‐authors of this article. To minimize bias, they were excluded from all editorial decision‐making related to the acceptance of this article for publication.

## ETHICS STATEMENT

All procedures followed were in accordance with the ethical standards of the responsible committee on human experimentation (institutional and national) and with the Helsinki Declaration of 1964 and its later amendments. This study was conducted in accordance with the Declaration of Helsinki, and approved by the Institutional Review Board of Saiseikai Shimonoseki General Hospital, and Yamaguchi University Hospital.

## CONSENT

Informed consent was obtained from all the patients in this study.
